# Skin infections due to Panton-Valentine leukocidin (PVL)-producing *S*. *aureus—*Cost effectiveness of outpatient treatment

**DOI:** 10.1371/journal.pone.0253633

**Published:** 2021-06-25

**Authors:** Marc-Nicolas Rentinck, Renate Krüger, Pia-Alice Hoppe, Daniel Humme, Michaela Niebank, Anna Pokrywka, Miriam Stegemann, Axel Kola, Leif Gunnar Hanitsch, Rasmus Leistner

**Affiliations:** 1 Institute of Hygiene and Environmental Medicine, Charité Universitätsmedizin Berlin, corporate member of Freie Universität Berlin, Humboldt-Universität zu Berlin and Berlin Institute of Health, Berlin, Germany; 2 Interdisciplinary workgroup on PVL-positive *S*. *aureus* (www.pvl-abszess.de), Charité Universitätsmedizin Berlin, corporate member of Freie Universität Berlin, Humboldt-Universität zu Berlin and Berlin Institute of Health, Berlin, Germany; 3 Division of Pulmonology, Immunology and Critical Care Medicine, Department of Pediatrics, Charité Universitätsmedizin Berlin, corporate member of Freie Universität Berlin, Humboldt-Universität zu Berlin and Berlin Institute of Health, Berlin, Germany; 4 Department of Dermatology, Venerology and Allergology, Charité Universitätsmedizin Berlin, corporate member of Freie Universität Berlin, Humboldt-Universität zu Berlin and Berlin Institute of Health, Berlin, Germany; 5 Department of Infectious Diseases and Respiratory Medicine, Charité Universitätsmedizin Berlin, corporate member of Freie Universität Berlin, Humboldt-Universität zu Berlin and Berlin Institute of Health, Berlin, Germany; 6 Institute of Medical Immunology, Charité Universitätsmedizin Berlin, corporate member of Freie Universität Berlin, Humboldt-Universität zu Berlin and Berlin Institute of Health, Berlin, Germany; 7 Division of Gastroenterology, Infectious Diseases and Rheumatology (including Nutritional Medicine), Medical Department, Campus Benjamin Franklin, Charité Universitätsmedizin Berlin, corporate member of Freie Universität Berlin, Humboldt-Universität zu Berlin and Berlin Institute of Health, Berlin, Germany; Leibniz-Institute DSMZ, GERMANY

## Abstract

**Introduction:**

Skin and soft tissue infections (SSTI) caused by Panton-Valentine leukocidin (PVL)-producing strains of *Staphylococcus aureus* (PVL-SA) are associated with recurrent skin abscesses. Secondary prevention, in conjunction with primary treatment of the infection, focuses on topical decolonization. Topical decolonization is a standard procedure in cases of recurrent PVL-SA skin infections and is recommended in international guidelines. However, this outpatient treatment is often not fully reimbursed by health insurance providers, which may interfere with successful PVL-SA decolonization.

**Aim:**

Our goal was to estimate the cost effectiveness of outpatient decolonization of patients with recurrent PVL-SA skin infections. We calculated the average cost of treatment for PVL-SA per outpatient decolonization procedure as well as per in-hospital stay.

**Methods:**

The study was conducted between 2014 and 2018 at a German tertiary care university hospital. The cohort analyzed was obtained from the hospital’s microbiology laboratory database. Data on medical costs, DRG-based diagnoses, and ICD-10 patient data was obtained from the hospital’s financial controlling department. We calculated the average cost of treatment for patients admitted for treatment of PVL-SA induced skin infections. The cost of outpatient treatment is based on the German regulations of drug prices for prescription drugs.

**Results:**

We analyzed a total of n = 466 swabs from n = 411 patients with recurrent skin infections suspected of carrying PVL-SA. PVL-SA was detected in 61.3% of all patients included in the study. Of those isolates, 80.6% were methicillin-susceptible, 19.4% methicillin-resistant. 89.8% of all patients were treated as outpatients. In 73.0% of inpatients colonized with PVL-SA the main diagnosis was SSTI. The median length of stay was 5.5 days for inpatients colonized with PVL-SA whose main diagnosis SSTI; the average cost was €2,283. The estimated costs per decolonization procedure in outpatients ranged from €50-€110, depending on the products used.

**Conclusion:**

Our data shows that outpatient decolonization offers a highly cost-effective secondary prevention strategy, which may prevent costly inpatient treatments. Therefore, health insurance companies should consider providing coverage of outpatient treatment of recurrent PVL-SA skin and soft tissue infections.

## Introduction

Skin and soft-tissue infections (SSTI) caused by Panton-Valentine leukocidin-producing *S*. *aureus* (PVL-SA) represent a significant burden for patients. The prevalence of community-acquired (CA) methicillin-resistant or susceptible *Staphylococcus aureus* (MRSA/ MSSA)-producing PVL varies worldwide [1,2]. The indication for screening is based on clinical presentation. In general, transmission is associated with multi-person households, contact sports [3], shared clothing [4] and steam baths [5]. International travel is another risk factor. This has been described, for example, in travelers returning from Africa [6] or the Americas [7].

The typical medical history shows recent first-time recurrent purulent skin abscesses of varying degrees of severity and without predilection site. Many cases show rapid dynamics of abscess development. It predominantly affects young, healthy patients with no pre-existing diseases. Differentially, PVL-SA can be distinguished, for example, from acne inversa, which tends to occur in the genital or axillary areas, or acne vulgaris, which has usually been present for a longer period of time [1,2].

This infection frequently leads to social stigmatization and eventually to significant psychological stress [1,2]. Fortunately, this pathogen rarely causes severe systemic infection and can be treated successfully in an outpatient setting [8–10]. In secondary prevention, following primary treatment, the nares and the entire surface of the body are decolonized topically using antiseptic agents [10,11]. There are no national guidelines in Germany for the examination of PVL production in clinically obtained *S*. *aureus* isolates. To facilitate the outpatient treatment of MRSA, reimbursement of the costs for MRSA screening, control swabs and decolonization therapy was introduced by the Joint Federal Committee (“Gemeinsamer Bundesausschuß”) in 2012 and has since been covered by the German statutory health insurance providers [12]. PVL-SA cases, however, were not included. In addition, it should be noted that despite decolonization recommendations in national and international guidelines [13,14], German health insurance providers only cover mupirocin nasal ointment for adult patients [12] and antiseptic gargle solution (compound e.g., Chlorhexidine 0.2%) for children 12 years of age or less [15]. We do not have internal data concerning reimbursement of PVL treatment at Charité Universitätsmedizin Berlin. However, with the exception of Italy, most other European countries (e.g. Austria, Hungary, France, Spain, the Netherlands and the Scandinavian countries) do not fully reimburse outpatient decolonization of MRSA-related infections [16]. It is therefore likely that PVL-SA treatment is not reimbursed in these countries either.

Decolonization measures cause a significant financial burden, particularly in multi-person households, since repeated decolonization procedures are often required [10]. Hence, the failure of German insurance providers to reimburse may discourage successful PVL-SA decolonization, especially in low-income households. Consequently, we set out to estimate the cost of inpatient treatment for PVL-SA in order to then evaluate the cost-effectiveness of outpatient decolonization as a preventive measure.

## Materials and methods

### Setting

The study was conducted at Charité Universitätsmedizin Berlin, a 3,000-bed tertiary care university hospital. The study period was January 1, 2014 to December 31, 2018. In 2015, our hospital established an interdisciplinary working group on PVL-SA infections. It operates several outpatient clinics where such patients are specifically treated. For this retrospective cohort study, we screened a total of 466 swabs from 411 patients (89.8% from outpatients and 10.2% from inpatients) for PVL-SA. 55 swabs were excluded due to repeated screening. Systematic sampling of selected patients did not take place. Patients with recurrent skin infections were tested for PVL-SA. The indication for screening was based on clinical presentation in patients with recurrent skin and soft tissue infection (SSTI) [1,2,10]. Although most PVL-SA skin infections are treated on an outpatient basis, some patients, for example severe cases or continued active infections despite repeated decolonization, were admitted for inpatient care. For our study, no specific follow up was made to monitor the success of decolonization for inpatients or outpatients.

**Fig 1:**
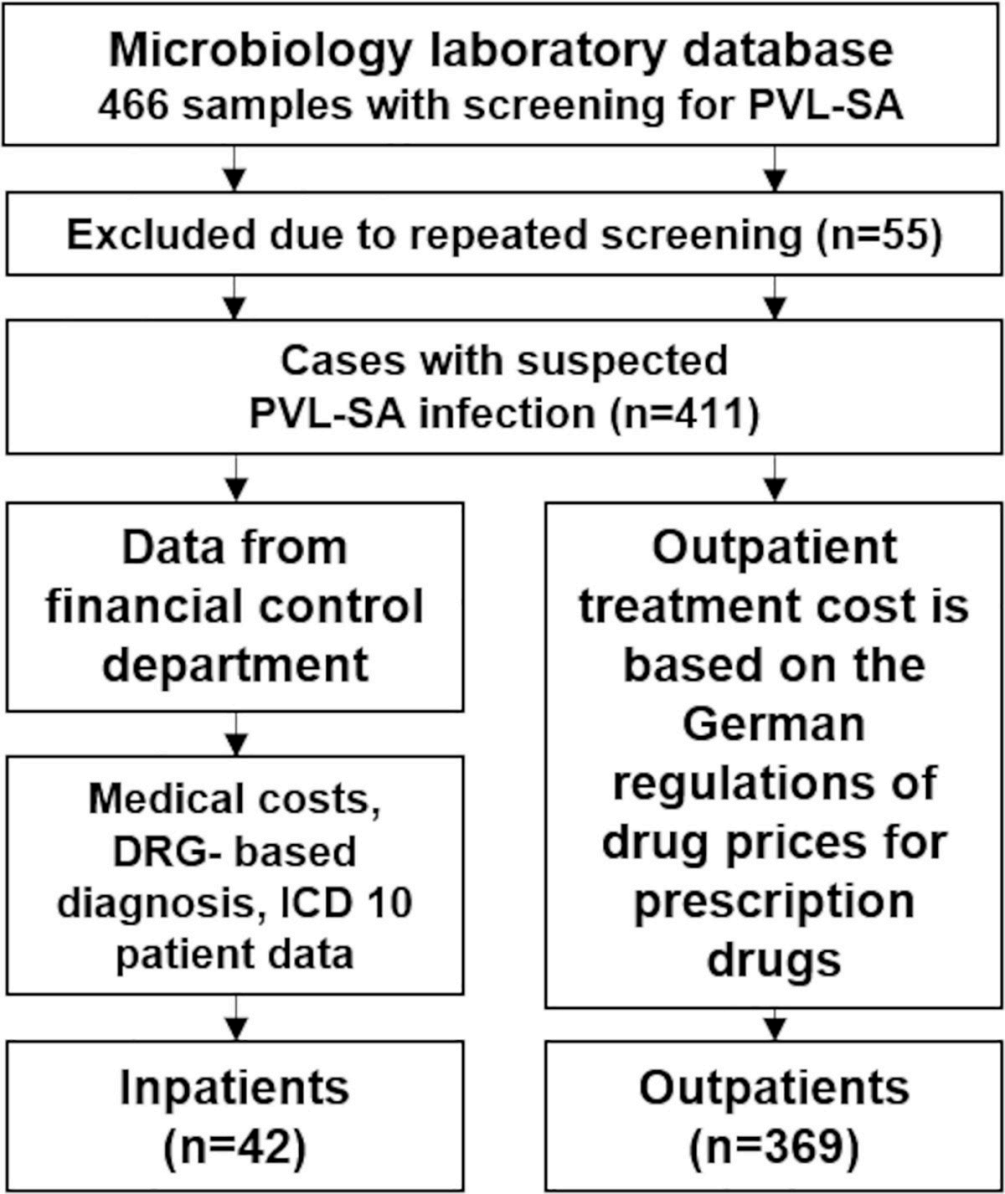
Overview data. PVL, Panton-Valentine leukocidin. SA, *Staphylococcus aureus*. DRG, Diagnosis Related Groups. ICD, International Classification of Diseases (Medical classification code for systematizing diagnoses).

The cohort analyzed was based on the extracted accumulated test results from our microbiology laboratory information system as of May 25, 2020. Further data on DRG (Diagnosis Related Groups)-based diagnoses and costs from the hospital perspective were provided by our financial controlling department. The cost of outpatient treatment is based on the German regulations of drug prices for prescription drugs (Fig 1).

The cohort of this study was subject to potential confounders, biases, and effect modifiers. These include swab sensitivity and colonization sites. In addition, patients with typical medical histories were studied, implying a high pretest probability.

In further addition, classification of our results in terms of prevalence as a function of MSSA versus MRSA was difficult due to biases in literature. These points will be addressed in more detail later in the discussion.

### Ethics approval

The study was conducted in accordance with the Declaration of Helsinki national and institutional standards. The study was approved by the local ethics committee (Charité, Berlin, Germany, EA2/190/17). The study was based on secondary data generated by routine clinical care. Within this scope, each patient provided written consent for treatment and secondary analysis. In the case of minors under the age of 18, consent was obtained from parents or guardians. The ethics committee waived the requirement for additional consent in this study.

### Microbiology

Swabs were taken from inpatients and outpatients being treated of Charité Universitätsmedizin Berlin and its facilities with corresponding symptoms indicative of PVL-SA. Indicative symptoms included recent first-time recurrent abscesses, which may manifest anywhere on the body and have a dynamic development of symptoms. The majority of patients were given a nasopharyngeal screening for PVL-SA. When applicable, samples were also taken from wounds, inguen and rectum. The swabs were analyzed in the bacteriological laboratory of the Institute of Hygiene and Environmental Medicine. Swabs were cultivated on Chrome Agar and Columbia Agar with 5% sheep blood (bioMérieux). Species identification and antimicrobial susceptibility testing were performed using a Vitek 2 system and applying EUCAST breakpoints. Gallery API ID 32 Staph (bioMérieux, Marcy l’Etoile, France) and the ID-GBP card of the VITEK 2 system (bioMérieux) were used following manufacturer recommendations for biochemical species identification. *S*. *aureus* isolates were confirmed using the isothermal DNA amplification of the Amplex system with the test kit eazyplex®MRSAplus (REF7611) to detect the MRSA-specific beta-lactamase genes mecA and mecC as well as the coding genes of the Panton-Valentine leukocidin (PVL) lukS and lukF [17].

### Financial parameters

Data concerning hospital costs for inpatients was derived from true hospital costs provided by the financial controlling department of the Charité Universitätsmedizin Berlin. Costs were broken down by total costs, total costs daily, costs for staff (medical/ nursing/technical), materials (pharmaceutical/ implant/ transplant/ other), medical/non-medical infrastructure (combined for staff and materials) following the official matrix standards of the German Institute for Hospital Fee Systems (Institut für das Entgeltsystem im Krankenhaus/ InEK). Costs were estimated based on the patient’s length of stay on each unit and distributed proportionally over the expenses for staff and material. Expenses that exceeded a certain cost threshold were actually included in the calculation (e.g., expensive artificial implants).

The cost of outpatient care medical products was estimated using data from the German prescription drug price regulations based on information from the WINAPO 64 pharmacy management tool database (CGM-Lauer, Koblenz, Germany; last accessed November 16, 2020).

### Statistics

Descriptive univariate analyses were performed for the entire cohort. The median and the interquartile ranges (IQR) were calculated for continuous parameters; number and percentage were calculated for binary parameters. Univariate differences were tested using the Wilcoxon rank-sum test for continuous variables and the Chi-square test for binary variables. All tests of significance were two-tailed with a p-value of <0.05 considered significant. All analyses were performed using SPSS (IBM SPSS statistics, Somer, NY, USA).

## Results

### Pathogen perspective

**Fig 2:**
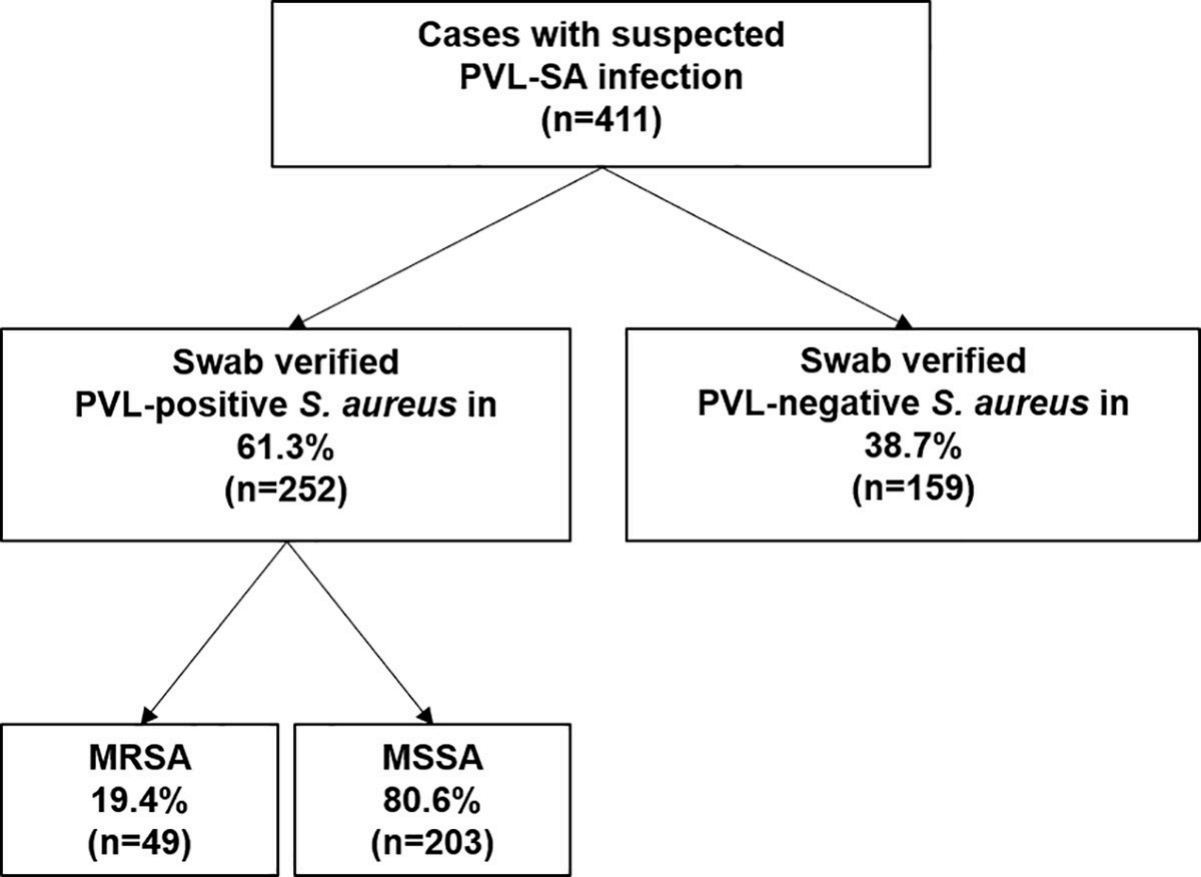
Overview recruitment pathogen perspective. PVL-SA isolated, stratified by methicillin-resistance (MRSA) and methicillin-susceptibility (MSSA). PVL, Panton-Valentine leukocidin. SA, *Staphylococcus aureus*.

Between January 1, 2014 and December 31, 2018, our laboratory received a total of n = 466 swabs from n = 411 patients suspected from their medical history and clinical presentation of having an infection involving PVL-SA. Overall, PVL-SA was detected in 61.3% of all patients included in the study. The majority (80.6%) of the isolates were methicillin-susceptible; 19.4% were methicillin-resistant *S*. *aureus* (MRSA) (Fig 2).

**Fig 3:**
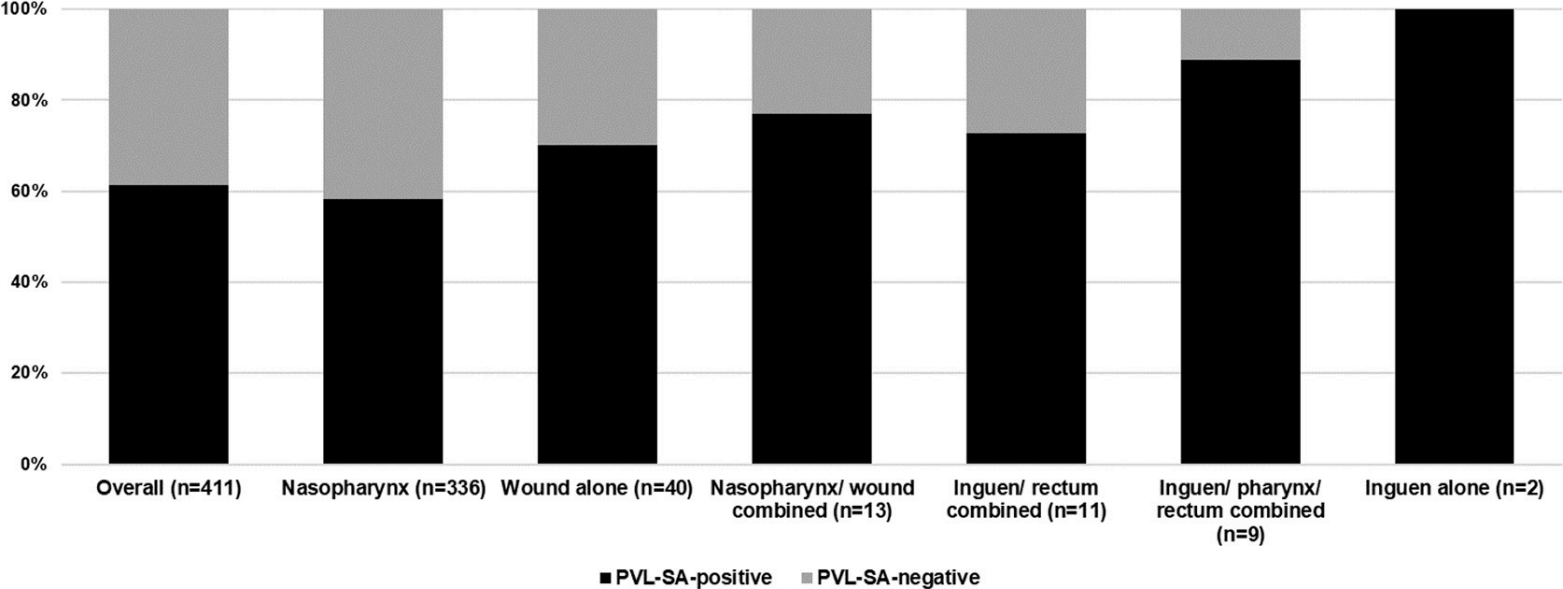
PVL-SA examination sites and associated detection rate. PVL-SA-positive, Panton-Valentine leukocidin *Staphylococcus aureus* positive. PVL-SA-negative, Panton-Valentine leukocidin *Staphylococcus aureus* negative. Pharyngeal, nasopharyngeal swab either combined or alone.

The most frequently examined sites were the nasopharynx, (n = 336), wounds (n = 40), combined nasopharynx / wound (n = 13), combined rectum/ inguen (n = 11), combined inguen/ pharynx/ rectum (n = 9), and inguen alone (n = 2) (Fig 3).

The patients in the MSSA and the MRSA sub-cohort did not differ significantly in median age in years (29.4 (IQR 13.5–38.3) and (27.4 (IQR 10.5–39.3) respectively, p-value = 0.889) or male gender (55.2% vs. 55.1%, p-value = 1.000) (not presented).

### Patient perspective

Of all cases (regardless of PVL-SA carriage), 89.8% were treated as outpatients (n = 369), 10.2% as inpatients (n = 42).

**Fig 4:**
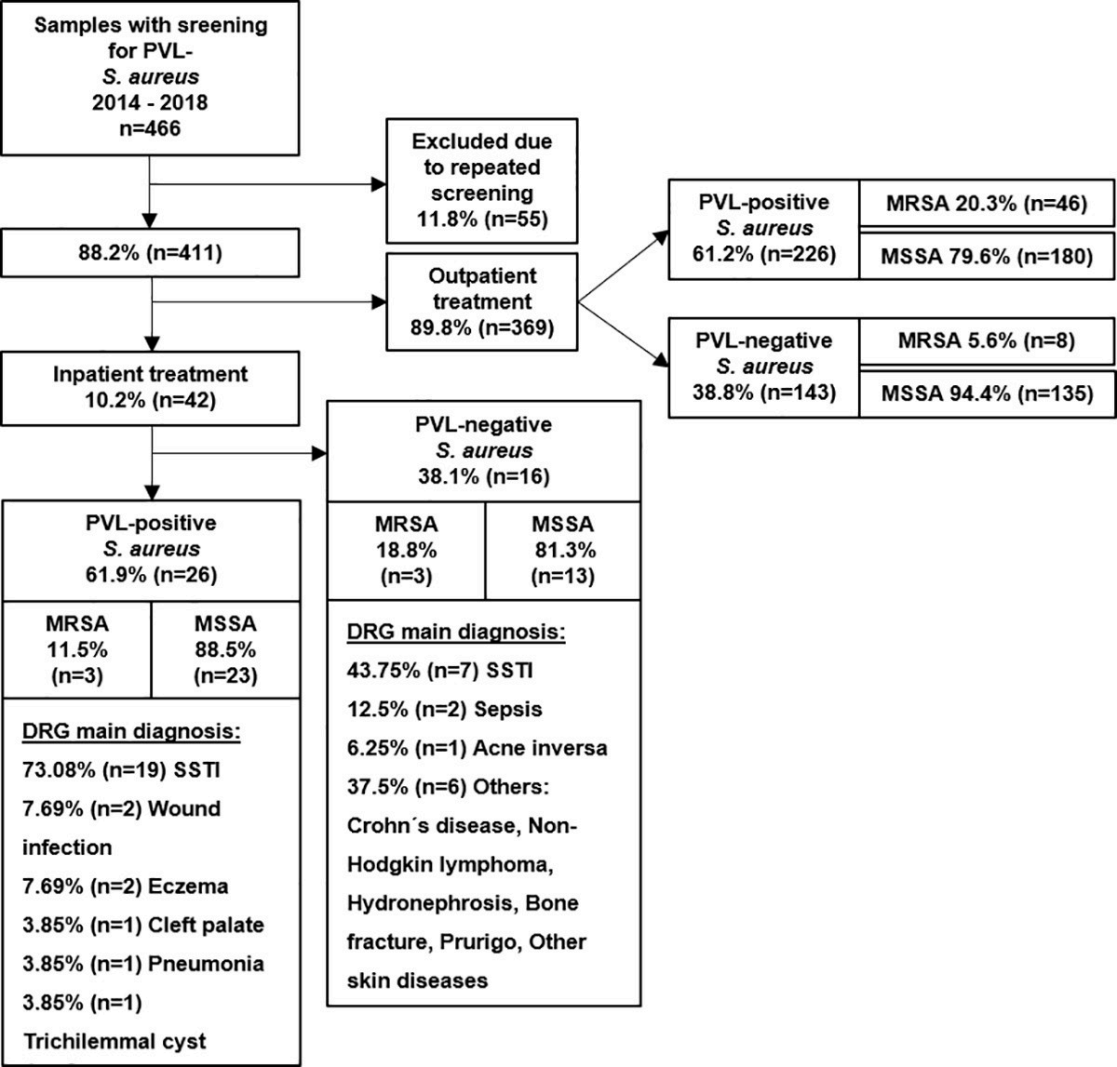
Overview recruitment patient perspective. MRSA, methicillin-resistant *Staphylococcus aureus*. MSSA, methicillin-susceptible *Staphylococcus aureus*. PVL, Panton-Valentine leukocidin. DRG, diagnosis related groups.

Of the inpatients, 61.9% (n = 26) were colonized with a PVL-positive *S*. *aureus* (PVL-pos-SA) and 38.1% (n = 16) with a PVL-negative *S*. *aureus* (PVL-neg-SA). Of all inpatients with the primary diagnosis SSTI (DRG main diagnosis), 73.1% (n = 19) were colonized with PVL-pos-SA and 26.9% (n = 7) were colonized with PVL-neg-SA. Of inpatients colonized with a PVL-pos-SA, SSTI was the primary reason for medical treatment for 73.0% (n = 19). Of inpatients colonized with a PVL-neg-SA, SSTI was the primary diagnosis for 43.8% (n = 7) (Fig 4).

Information on the primary DRG diagnosis was not available for outpatients. However, outpatients were specifically treated in our PVL-SA outpatient clinic and presented either recurrent SSTI or SSTI associated with a PVL-SA outbreak. Even though, not statistically significant, inpatients were more often male and slightly older than outpatients (Table 1). PVL-SA positive inpatients whose primary diagnosis was SSTI had a median length of stay of 5.5 days (IQR 4.0–7.9) and total median costs of €2283.10 (IQR €1851-€3856.4) (Table 2).

**Table 1:** Comparison of basic epidemiological parameters for inpatients and outpatients.

Parameters	Outpatients (n = 369)	Inpatiens (n = 42)	P-value
**Age (years)**	27.6 (9.8–38.9)	32.4 (22.3–48.7)	0.284
**Male gender**	50.1% (n = 185)	64.3% (n = 27)	0.085
**MRSA**	14.6% (n = 54)	14.3% (n = 6)	0.986
**PVL-positive**	61.2% (n = 226)	61.9% (n = 26)	0.943

**Table 2:** Financial parameters of inpatient treatment of PVL-SA infections with SSTI as the primary diagnosis.

Financial parameters of n = 19 PVL-SA pos inpatients with SSTI as the primary diagnosis		
Parameter		PVL-positive
LOS Total		5.5 (4–7.9)
LOS Normal ward		5.5 (3.9–7.9)
LOS Intensive care unit		0 (0–0)
**Costs in EURO (€)**	Total	2283.1 (1851–3856.4)
	Daily	443.8 (372–504.3)
	Medical staff	381.2 (291.9–650.1)
	Nursing staff	641.3 (526–869.3)
	Technical staff	143.5 (76.9–220.7)
	Pharmaceutical materials ^1^	59.1 (43–90.1)
	Pharmaceutical materials ^2^	0 (0–1.5)
	Implant/transplant materials	0 (0–0)
	Other medical materials ^1^	66.5 (38.7–142.8)
	Other medical materials ^2^	4 (0–52.1)
	Medical infrastructure (staff and materials)	131.5 (60.7–170.5)
	Non-medical infrastructure (staff and materials)	362.6 (222–733.9)

LOS, length of stay. PVL, Panton-Valentine leukocidin. SA, *Staphylococcus aureus*.

^1^ Costs estimated by totaling of all costs in this category per ward and estimated proportionally to individual length of stay.

^2^ Actual costs per article above a predefined threshold.

Concerning outpatient costs, we estimated the average cost per decolonization procedure. One decolonization procedure was equivalent to a 5-day treatment. Outpatient costs were estimated using official information from the German drug price regulations for prescription drugs (Access date: November, 16^th^ 2020) (S1 Table). For example, a 5-day treatment course, for a patient, based on our experience, required 1 tube of mupirocin nasal ointment (3g), 200-300ml of mouthwash and gargle solution (compound e.g., Chlorhexidine 0.2%), 1,000ml of whole body washing lotion (compound e.g., Octenidin), 500ml of hand disinfectant (compound e.g., disinfection based on ethanol) and 1,000ml of surface disinfectant (compound e.g., rapid disinfection based on ethanol).

Mupirocin is the only product that is reimbursed by the health insurance providers in Germany. There are many similar products available over the counter without strict price regulation. Thus, it was difficult to estimate an exact price overall. We combined several possible products and ended up with an average price of about €50. However, complete decolonization kits are available for approximately €110 (S1 Table).

A previous study has showed that up to 5 decolonization procedures can be necessary to achieve a success rate of 89% [10]. Therefore, calculated together with our cost estimate, costs could total as much as €250-€550. However, not included in this estimate are other costs, such as washing clothes, the disposal of tooth brushes, hair brushes, and essentially all personal hygiene products. These additional costs are difficult to determine but will likely be significant.

## Discussion

We estimated that the current cost of a single outpatient decolonization procedure ranges from €50 to €110 for a single patient. Thus, for a maximum of 5 repetitions, the total cost would be approximately €250-€550, depending on the products chosen. In contrast, according to our analysis, at a median PVL-SA inpatient cost of €2,283, the cost of treating inpatients with a primary diagnosis of SSTI was on average 5–10 times higher. In addition, after discharge former inpatients have to pay out of pocket for all decolonization procedures performed on an outpatient basis as they are not currently reimbursed fully by health insurance providers in Germany. Early diagnosis of PVL-SA-related infections and subsequent outpatient decolonization of patients and their close contacts could prevent severe infections that would require costly inpatient treatment as well as conserving hospital resources.

During the course of this study, additional secondary points have emerged that might influence the treatment of PVL-SA induced skin infections.

### Sensitivity swab and colonization site

The overall detection rate of the PVL-SA examination in our cohort was 61.3%. We observed increased detection rates when we combined swabs from different sites when examining individual patients. This illustrates the importance of carefully planned sampling. The sensitivity of *S*. *aureus* swabs in general has been relatively well researched [18]. About 20% of the general population are permanent nasal carriers of *S*. *aureus* while 30% are intermittent carriers [18]. Furthermore, earlier studies have shown that humans can be colonized simultaneously by more than one *S*. *aureus* strain [19]. Thus, various *S*. *aureus* strains may well grow on a single agar plate, of which some may be PVL-positive, others PVL-negative. In PVL-SA detection, a standard clinical laboratory does not analyze all of the colonies on the agar plate for PVL genes, but only a few of them. As a result, it is possible for the target analyte to be overlooked in routine sampling. This overlook effect has also been demonstrated in the whole-genome sequencing performed in ESBL- *E*. *coli* (extended-spectrum-beta-lactamase-producing *Escherichia coli*) research [20]. The reliable determination of PVL-SA helps reduce its spread and to prevent extending patient suffering unnecessarily. Hence, this may be an area in the field of PVL-SA worth investigating in the future.

### Main admission diagnosis SSTI

We had higher detection rates in patients with SSTI as the primary diagnosis than in patients with a different primary diagnosis. This indicates a higher pretest probability in patients with a clinically typical medical history, for example recurrent SSTI. This is known from earlier studies [1]. However, 39% of our outpatients and 27% of our SSTI inpatients tested negative for PVL-SA. This could be due to an alternative causality (e.g., PVL-negative SA) or to the low sensitivity of the swab. Test results play an important role in the decision process concerning PVL-SA outbreaks and treatment. Therefore, concrete studies are needed that address the negative predictive value in cases with a high pretest probability.

### MRSA vs. MSSA

The average rate of PVL-SA colonization in the general population is not known. A few studies have been done in various countries that address this topic, but there are factors that render their results prone to distortion [21,22]. The most relevant bias is the fact that most available studies focus on PVL-positive MRSA, often termed community-acquired MRSA and primarily associated with the clone USA-300. Several European studies, in contrast, have shown that most clinically relevant strains of SSTIs (60–90%) are methicillin-susceptible [10,17,23]. A further limitation of the published studies is the pre-selection of patients, e.g., travelers, MRSA carriers, hospital patients, which limits general comparability. The data for our study region (Berlin und Brandenburg, Germany) confirms that most SSTIs that cause PVL-SA are MSSA. Nevertheless, our study showed that the MRSA infection rate among PVL-SA-induced skin infections was relatively high at 19.4%. This indicates, when compared with the overall nasal MRSA colonization rate of approximately 1–3% [11], that there might be PVL-positive MRSA subpopulations associated with relevantly higher risk of SSTI. Future studies addressing the average prevalence of PVL-SA among the general population should focus primarily on PVL-SA in general and only secondarily on methicillin-resistance.

### Limitations

This study has limitations. First, the data at hand was derived from a retrospective study. We did not have the real data on the costs of outpatient decolonization and thus had to estimate those costs based on the current literature and German regulations of the price of prescription drugs. Despite costs being specific to Germany, the cost ratio between inpatients and outpatients is likely similar in other countries, with the result that outpatient decolonization is cost effective in general. We did not perform a follow up of the successful outcome of decolonization of the inpatients and outpatients. The final cost of inpatient as well as outpatient treatment can, therefore, not be established conclusively. However, during the study period no patients were readmitted with the same diagnosis.

## Conclusion

Our study showed that a single inpatient treatment procedure for PVL-SA SSTI was on average five to ten times more expensive than successful outpatient decolonization. Hence, we conclude that outpatient decolonization is cost-effective for secondary prevention of PVL-SA-associated SSTI. Considering that such a procedure apparently reduces both the health and financial burden of affected patients, health insurance providers should consider covering this treatment of verified PVL-SA infections.

## Supporting information

S1 Table Outpatient costs estimated on the official information of the German drug price regulation for prescription drugs. PZN (Pharmacy Central Number), a Germany-wide identification key for pharmaceuticals, medical devices and other products commonly used in pharmacies.
(PDF)

S1 Database Anonymized original dataset. (XLSX)
