# Supplementary material for: Skin infections due to Panton-Valentine leukocidin (PVL)-producing S. aureus—Cost effectiveness of outpatient treatment
Source: PLoS One. 2021 Jun 25;16(6):e0253633. doi: 10.1371/journal.pone.0253633 (PMC8232536; doi:10.1371/journal.pone.0253633)
Supplement: S1 Table — PZN (Pharmacy Central Number), a Germany-wide identification key for pharmaceuticals, medical devices and other products commonly used in pharmacies. (PDF) [file pone.0253633.s001.pdf]

S1 Table. Outpatient costs estimated on the official information of the German drug price regulation for prescription drugs.

Prices were requested on November 16, 2020. Prices are as of November 15, 2020. Program WINAPO 64 (CGM-Lauer, Koblenz, Germany).

**PZN** (Pharmacy central number)	**Product name**	**Amount**	**Provider**	**Actual selling price (€)**	
16769211	Bactroban Nasal Ointment	3g	10CA	29,12	**Prescription and reimbursable**
09175681	Bactroban Nasal Ointment	3g	ACAMU	29,12	
10090369	Bactroban Nasal Ointment	3g	ALLOB	28,84	
12486568	Bactroban Nasal Ointment	3g	ALPH	33,05	
06307521	Bactroban Nasal Ointment	3g	AXICP	29,07	
11562190	Bactroban Nasal Ointment	3g	BERAG	31,40	
11102548	Bactroban Nasal Ointment	3g	CCPHA	29,19	
02171651	Bactroban Nasal Ointment	3g	EMRA	29,72	
04112428	Bactroban Nasal Ointment	3g	EURIM	29,19	
01499800	Bactroban Nasal Ointment	3g	KOHL	35,59	
05559870	Bactroban Nasal Ointment	3g	ORI	29,12	
01925071	Bactroban Nasal Ointment	3g	GERKE	23,10	
**3g Mupirocin per 5-day treatment**					
15393301	Mupirocin InfectoPharm 20 mg/g Nasal Ointment	5g	INFEC	41,32	
04524898	Turixin Nasal Ointment 20mg/g 2%	3g	GSK	44,69	
**Chlorhexidine digluconate (One measuring cup is approx. 10ml. If one assumes two units per day, this will last 10-15 days)**					**Not subject to prescription**
01208375	Chlorhexidine digluconate Ready solution 0.2%. Mouthwash and gargle solution	200ml	Engelhard Arzneimittel	7,22	
12574692	Chlorhexamed Forte alcohol free 0.2% Mouthwash and gargle solution	300ml	GSK	14,04	
**Sterilium classic pure as hand sanitizer (disinfection based on ethanol)**					
04817436	Sterilium classic pure	100ml	Hartmann	2,09	
04817465	Sterilium classic pure	500ml	Hartmann	6,79	

Octenisan washing lotion (whole body, compount Octenidin), 1 l is sufficient for 5 days (1 treatment)

02840537	Octenisan washing lotion	150ml	SCHÜLKE	2,99
02840543	Octenisan washing lotion	450ml	SCHÜLKE	3,69
05702770	Octenisan washing lotion	500ml	SCHÜLKE	5,10
02840566	Octenisan washing lotion	1l	SCHÜLKE	7,43

Octenisept solution (compount Octenidin) for the treatment of wounds etc.

03853387	Octenisept solution	250ml	SCHÜLKE	12,81
05702764	Octenisept solution	500ml	SCHÜLKE	18,91

Descosept sensitive wipes and solution as surface desinfectant (rapid disinfection based on ethanol)

14307328	Descosept sensitive wipes	100 pieces	SCHUM	8,30
01898718	Descosept pur	1l	SCHUM	6,20

Decolonization kits for head-to-toe decolonization according to the recommendations of the RKI. (The Prontoderm series of BRAUN also offers various other individual products for decolonization)

01048612	**Prontoderm MRSA Kit**	1 unit	BRAUN	106,17
**Kit contains:**				
04398229	Prontoderm solution (full body wash)	4 x 500ml	BRAUN	
00795270	ProntOral Mouthwash	1 x 250ml	BRAUN	
04398270	Prontoderm Foam (cleansing foam for hair and mucous membrane)	1 x 200ml	BRAUN	
04398264	Prontoderm Nasal Gel	1 x 30ml	BRAUN	
09721273	**Prontoderm MRE hygiene set**	1 unit	BRAUN	**No price found**
**Kit contains:**				
04398229	Prontoderm solution (full body wash)	1 x 500ml	BRAUN	
04398270	Prontoderm Foam	1 x 200ml	BRAUN	
00795270	ProntOral Mouthwash	1 x 250ml	BRAUN	
04398264	Prontoderm Nasal Gel	1 x 30ml	BRAUN	
01980466	Softa-Man Visco-Rub	1 x 100ml	BRAUN	
01889406	Meliseptol HBV Wipes	1 tin	BRAUN	
	Disposable combs			
	Disposable toothbrushes			

Not subject to prescription
